# Functional interaction of H_2_-receptors and 5HT_4_-receptors in atrial tissues isolated from double transgenic mice and from human patients

**DOI:** 10.1007/s00210-021-02145-8

**Published:** 2021-09-25

**Authors:** Joachim Neumann, Denise Schwarzer, Charlotte Fehse, Rebecca Schwarz, Margareta Marusakova, Uwe Kirchhefer, Britt Hofmann, Ulrich Gergs

**Affiliations:** 1 Institut Für Pharmakologie Und Toxikologie, Martin-Luther-Universität Halle-Wittenberg, Medizinische FakultätMagdeburger Str. 4, 06112 Halle, Germany; 2 Department of Pharmacology and Toxicology, Faculty of Pharmacy, Comenius University in Bratislava, Bratislava, Slovakia; 3 Institut Für Pharmakologie Und Toxikologie, Westfälische Wilhelms-Universität, Medizinische FakultätDomagkstr. 12, 48149 Münster, Germany; 4 Cardiac Surgery, Martin-Luther-Universität Halle-Wittenberg, Medizinische Fakultät, 06097 Halle, Germany

**Keywords:** Serotonin, Histamine, Inotropy, Chronotropy, Transgenic mice, Human atrium, H_2_-histamine receptor, 5-HT4-receptors, Heart

## Abstract

In the past, we generated transgenic mice that overexpress the human histamine 2 (H_2_)-receptor (H_2_-TG) or that overexpress the human serotonin 4 (5-HT_4_)-receptor (5-HT_4_-TG) in the heart. Here, we crossbred these lines of mice to generate double transgenic mice that overexpress both receptors (DT). This was done to study a conceivable interaction between these receptors in the mouse heart as a model for the human heart. When in left atria, initially, force of contraction was elevated maximally with 1 µM serotonin, and subsequently, histamine was cumulatively applied; a biphasic effect of histamine was noted: the force of contraction initially decreased, maximally at 10 nM histamine, and thereafter, the force of contraction increased again at 1 µM histamine. Notably, functional interaction between 5-HT and histamine was also identified in isolated electrically stimulated trabeculae carneae from human right atrium (obtained during cardiac surgery). These functional and biochemical data together are consistent with a joint overexpression of inotropically active H_2_-receptors and 5-HT_4_-receptors in the same mouse heart. We also describe an antagonistic interaction on the force of contraction of both receptors in the mouse atrium (DT) and in the human atrial muscle strips. We speculate that via this interaction, histamine might act as a “brake” on the cardiac actions of 5-HT via inhibitory GTP-binding proteins acting on the activity of adenylyl cyclase.

**Supplementary Information:**

The online version contains supplementary material available at 10.1007/s00210-021-02145-8.

## Introduction

Cardiac contractile effects of histamine in man are explained by a direct activation of histamine receptors in cardiac cells of the heart. Four different G-protein coupled heptahelical histamine receptors, the H_1_-, H_2_-, H_3_-, and H_4_-histamine receptors, have been described (Jutel et al. 2009). The H_1_-, H_3_-, and H_4_-receptors can stimulate the enzymatic activity of phospholipase C (PLC) and/or can inhibit adenylyl cyclase activity (review: Panula et al. 2015), whereas the H_2_-receptors can activate adenylyl cyclase activity (human heart: Klein and Levey 1971, Bristow et. al. 1982a, b). Histamine shows regional functional differences with respect to force generation (atrium vs. ventricle) and species differences in its cardiac actions.

**Fig. 1:**
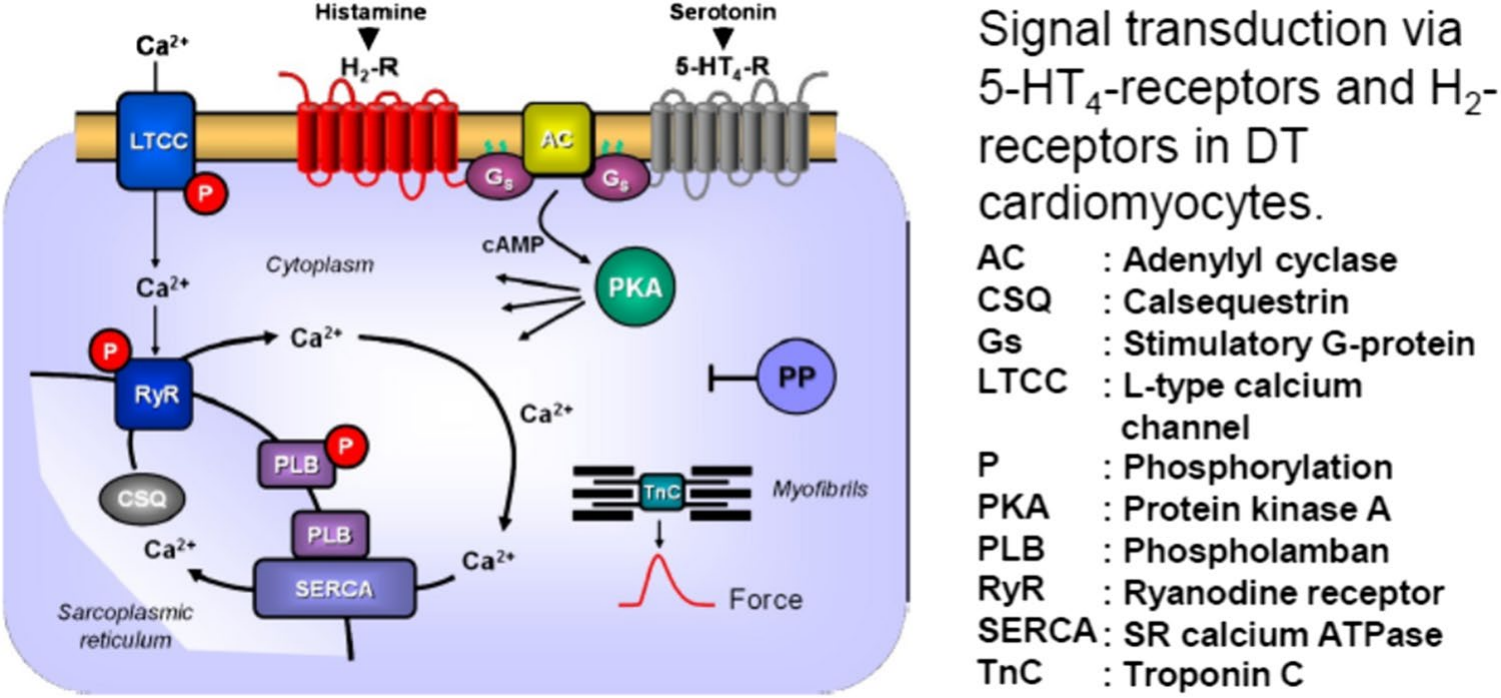
A scheme of a cardiomyocyte: histamine and serotonin bind from the outside to sarcolemmal H_2_-receptor and 5-HT_4_-receptors, respectively, the occupation of which by histamine or serotonin increases the enzymatic activity of adenylyl cyclase (AC) in the sarcolemma of heart muscle cells brought about by stimulatory G-proteins (Gs). This augments the subsequent production of cAMP and, thereby, activates cAMP-dependent protein kinase (PKA). PKA increases cardiac force generation and relaxation by increasing the phosphorylation state (P) of, for instance, the L-type Ca^2+^ channel (LTCC), phospholamban (PLB), the ryanodine receptor (RYR), and other regulatory proteins, not depicted here. These increases in the phosphorylation state of proteins are reversed by proteins phosphatases (PP). Trigger Ca^2+^ passing through the LTCC initiates release of Ca^2+^ from the sarcoplasmic reticulum via RYR into the cytosol, where Ca^2+^ activates myofilaments and leads to increased force generation. When the heart relaxes, Ca^2+^ is taken up into the sarcoplasmic reticulum via a sarcoplasmic reticulum Ca^2+^ ATPase (SERCA) when the phosphorylation state of PLB is elevated by PKA on serine 16 or by CAM kinase on threonine 17. Not shown: receptors may also act in part, on inhibitory G-proteins thereby inhibiting AC which was previously stimulated by another G-protein coupled receptor (like the β-adrenoceptor) reducing cAMP content and thence force of contraction

For instance, in the rabbit atrium and ventricle, the H_1_- and H_2_-receptors are both expressed at the RNA and protein levels. In the rabbit heart, H_2_-receptors mediate the positive inotropic and chronotropic effects of histamine in atria, whereas H_1_-receptors are predominantly involved in the positive inotropic effect of histamine in ventricles (Hattori et al. 1988, 1990, 1991). Likewise, in humans, the H_1_- and H_2_-histamine receptors were detected several decades ago in the atrium and ventricle (radioligand binding: Baumann et al. 1982, 1983, 1984, antibody and mRNA expression: Matsuda et al. 2004). In humans, the cardiac H_2_-receptors were thought to mediate the PIE and PCE of exogenously applied histamine in isolated human cardiac preparations (atrium: Levi et al. 1981, Genovese et al. 1988, Zerkowski et al. 1993, Sanders et al. 1996, ventricle: Ginsburg et al. 1980). The PIE of histamine in the human heart was accompanied (and hence probably mediated) by an increase in the 3′,5′-cyclic adenosine monophosphate (cAMP) content, by activation of cAMP-dependent protein kinase (PKA, human right atrial preparations: Sanders et al. 1996), and by an increased current through L-type Ca^2+^ channels (ventricle: Eckel et al. 1982, compare scheme in Fig. 1); these responses then led to an increase in the phosphorylation state of phospholamban (Gergs et al. 2019a). Hence, the mode of action of H_2_-receptors in the human heart mimics that of the β-adrenoceptor system.

Moreover, isolated preparations from human atria show a PIE and a relaxant (or lusitropic) effect when treated with serotonin (5-HT) (Kaumann, 1990; Kaumann and Sanders, 1994; Sanders and Kaumann 1992). Under normal conditions, 5-HT is stored in thrombocytes, so the heart receives 5-HT from the blood (Verbeuren 1990; 1992). In the past, we presented evidence that 5-HT and histamine can also be produced by and found in considerable amounts in isolated cardiomyocytes (Neumann et al. 2021, Pönicke et al. 2012). The cardiovascular effects of 5-HT have been reviewed in detail elsewhere (Kaumann and Levy, 2006, Neumann et al. 2017).

The PIE response to 5-HT includes an increase in cAMP-content and an increase in the activity of protein kinase A (PKA). In addition, in cardiomyocytes from human heart, 5-HT treatment increased the currents through L-type Ca^2+^-channels due to increased channel availability (Jahnel et al. 1992, 1993) and the phosphorylation state of phospholamban (Gergs et al. 2009). The lusitropic cardiac effects of cAMP-increasing drugs like 5-HT are usually explained by this phospholamban phosphorylation. The reversal of the PIEs of 5-HT in human cardiac preparations by specific 5-HT_4_ antagonists led to the conclusion that those PIEs were mediated by 5-HT_4_ receptors (Sanders et al., 1995).

In isolated pig heart preparations and in anaesthetized pigs, 5-HT has shown a positive chronotropic effect (Kaumann 1990; Villalón et al. 1990). This porcine effect was also classified as 5-HT_4_-receptor-mediated, based on antagonist studies (Kaumann 1990). Interestingly, the pig seems to be the only species besides man that exhibits a PIE and a PCE that is induced by 5-HT activation of 5-HT_4_-receptors (Kaumann 1990). Other animal species show PIE or PCE in response to 5-HT, but their effects are not mediated by 5-HT_4_-receptors but via a release of endogenous noradrenaline (overview: Kaumann and Levy 2006, Neumann et al. 2017). In the rabbit heart, serotonin is not only taken up into cardiac sympathetic nerves and releases noradrenaline from the storage sites but also acts as an endogenous β-adrenoceptor antagonist (Hattori et al. 1992).

The PIE of histamine is mediated by H_1_-receptors in the porcine ventricle, whereas the PIE of histamine in human ventricles is mainly mediated by H_2_-receptors (Du et al. 1993). Hence, an animal model that expresses both functional human 5-HT_4_-receptors and human H_2_-receptors in the ventricle and the atrium is currently lacking and would be a relevant and a convenient model for preparations from the human myocardium. The aim of this study was to develop this type of model.

Isolated mouse (WT; wild-type = non-transgenic) cardiac preparations show no response to 5-HT or histamine (with regard to inotropy and chronotropy), presumably because of the lack of receptor protein expression or lack of effector coupling (Gergs et al. 2010, 2013, 2019a). We have generated suitable models for these human receptors in our previous studies by producing transgenic mice that overexpress the human 5-HT_4a_ receptor (5-HT_4_-TG) or the human H_2_ receptor (H_2_-TG mice) only in the heart by means of a cardiac-specific promoter sequence. In these 5-HT_4_-TG hearts (but not in WT hearts), 5-HT exerts both a PIE and a PCE (Gergs et al. 2010, 2013), while in the H_2_-TG mice, histamine exerts a PIE and a PCE (Gergs et al. 2019a, 2020).

The present study was initiated to determine whether the inotropic and chronotropic effects of histamine and serotonin are detectable and show functional contractile activity in intact heart, isolated hearts and cardiac atrial preparation from double transgenic (DT) mice engineered to express both the human H_2_-receptor and the 5-HT_4_-receptor, as in the human heart. A second aim was to determine whether interactions occurred between 5-HT and histamine with respect inotropy occur in DT and to ask whether the same interactions are also detectable in the human heart and might therefore have clinical relevance.

Parts of this investigation have been published before in abstract form (Schwarzer et al. 2019; Neumann et al. 2019).

## Materials and methods

### Transgenic mice

Transgenic mice (TG) with cardiac myocyte-specific overexpression of the human 5-HT_4_ receptor or the H_2_ receptors and their littermate control mice (WT) were generated as described by Gergs et al. (2010, 2019a). Both lines were crossbred to obtain double transgenic mice (DT). Heart-specific expression was achieved via the α-myosin heavy-chain promoter. The age of the animals ranged from three to five months. Animals were handled and maintained according to approved protocols of the animal welfare committee of the University of Halle-Wittenberg, Halle.

### Contractile studies in mice

In brief, mice were sacrificed, the thorax was opened, the heart was mobilized and cut from the ascending aorta to make sure the right atrium was not damaged. Then, the whole heart was transferred to a dissection chamber filled with gassed Tyrode’s solution at room temperature. Right or left atrial preparations were isolated and mounted in organ baths at 37 °C with a as described by Gergs et al. (2013, 2017, 2019b) and Neumann et al. (2003). The atrial preparations were stimulated by rectangular pulses (5 ms duration and voltage 10% of threshold) of 1 Hz frequency. The right atrial preparations were allowed to beat spontaneously. Force was detected under isometric conditions, amplified and fed into a digitizer and quantified by a commercial software (Chart 5, Adistruments, Oxford, United Kingdom).

### Contraction studies in human atrium

This was performed as reported repeatedly, e.g., by Gergs et al. (2009). In brief, during cardiac surgery, at the site where the cannula for extracorporeal circulation entered the heart, small muscle strips were obtained from the right atrium. Patients were aged between 48 and 72 years and their written informed consent was obtained for the use of their right atrial tissues prior to undergoing cardiac surgery. Medication included acetylsalicylic acid, nitrates, diuretics, β-adrenoceptor blockers and anticoagulants. Trabeculae were dissected and mounted in organ baths and electrically stimulated (1 Hz) (5 ms duration and voltage 10% of threshold) at 37 °C and force recordings were processed like in mouse atrial preparations.

### Western blotting

The processes of sample homogenization, protein concentration measurement, electrophoresis, antibody incubation and signal quantification were performed following our previously published protocols (Gergs et al. 2009, 2019a, 2019b; Boknik et al. 2018). In the past, we it was necessary to prepare membranes enriched in the sarcoplasmic reticulum to detect phospholamban (PLB) and its phosphorylation state in radioactively labeled isolated hearts (for example: Neumann et al. 1993). More recently, an antibody against phosphorylated PLB has become commercially available, where one does not need to prepare membranes in atrial or ventricular preparations but where cardiac homogenates are sufficient (for example: Gergs et al. 2009).

Electrophoresis was performed in Novex™ 4–12% “Tris–Glycine Plus Midi Protein Gels” (Invitrogen, Thermo Fisher Scientific, Waltham, Massachusetts, USA), The run was performed at 4 °C for approximately 1 h at 120 V in the “NuPAGE MES SDS Running Buffer” (Thermo Fisher Scientific, Waltham, Massachusetts, USA) using the Bio-Rad system (Bio-Rad Laboratories, Hercules, California, USA). Protein transfer into membranes (Amersham™ Protran, GE Healthcare, Chicago, IL, USA) was performed at 2 A for 2 h at 4 °C. Membrane blocking for 1 h at room temperature was followed by overnight incubation at 4 °C with the primary antibody for serine 16—phosphorylated phospholamban (catalogue number: A010-12AP; PLB Ser16, Badrilla, Leeds, UK), while calsequestrin antibody (CSQ2) was used as loading control (product number: ab3516; abcam, Cambridge, UK). Visualization of the signal was performed by using enhanced chemifluorescence staining (“ECF™ Substrate for Western Blotting,” Amersham, GE Healthcare, Chicago, IL, USA) and a Typhoon 9410 Imager (GE Healthcare, Chicago, IL, USA). Quantification was performed using ImageQuant TL image analysis software (GE Healthcare, Chicago, IL, USA).

### Echocardiography

Echocardiography was performed as published previously (Boknik et al. 2019, Gergs et al. 2018). After induction of anaesthesia by isoflurane (Forene®, AbbVie, North Chicago, IL, USA), 100 µl of 1 mM 5-HT solution (5-hydroxytryptamine (serotonin) hydrochloride, Lot. 121K7059, Sigma-Aldrich Chemie GmbH, Germany) or the same volume of a 1 mM solution of histamine (histamine dihydrochloride, EC No. 2002984, Fluka BioChemika, St. Gallen, Switzerland) were injected. Cardiac left ventricles were visualized using the Vevo 2100 Linear Imaging System (VisualSonics Inc., Toronto, Ontario, Canada).

### Real-time polymerase chain reaction

Real-time polymerase chain reaction (PCR) analysis was performed as described previously (Gergs et al., 2019a, b, c). Total RNA from cardiac ventricular samples was isolated by phenol/chloroform extraction (TRI Reagent®, Cat. 15,596,026, Invitrogen, Thermo Fisher Scientific, Waltham, Massachusetts, USA) and transcribed into cDNA via “Maxima First Strand cDNA Synthesis Kit” (Lot. 00,959,956, Thermo Fisher Scientific, Waltham, Massachusetts, USA). Real-time PCR (Bio-Rad CFX Connect cycler, Bio-Rad Laboratories, Hercules, California, USA) was performed using “iTag Universal SYBR Green Supermix” (Cat. 1,725,121, Bio-Rad Laboratories, Hercules, California, USA). Glyceraldehyde 3-phosphate dehydrogenase (GAPDH) was used as a reference gene for calculations of target gene expressions, namely human and mouse 5-HT_4_- and H_2_-receptors (for primer sequences see Table 3). Relative expression of mRNA was calculated by 2^−∆∆Ct^ method (Livak and Schmittgen, 2001).

### Langendorff perfusion

Hearts were isolated and retrogradely perfused in a custom made glass perfusion system at 37 °C following our own procedures (Gergs et al. 2010, 2017, 2019b). Force was measured under isometric conditions from the apex cordis, amplified and digitized. At the peak of the contractile effects (5 min), whole hearts were shock frozen with aluminum clamps (Wollenberger clamps) previously cooled in liquid nitrogen and kept at -80 °C until further analysis.

### Data analysis

Data were treated as in most our previous studies (e.g. Gergs et al. 2019a, b, c). Shown are means ± standard error of the mean. Statistical significance was estimated by analysis of variance followed by Bonferroni’s *t*-test. A *P*-value of less than 0.05 was considered significant. Experimental data for agonist-induced positive inotropic and chronotropic effects were analyzed by fitting sigmoidal curves to the experimental data with GraphPad Prism 5.0. All other statistical analyses were performed as indicated in the figures and tables.

### Drugs and materials

(-)-Isoprenaline ( +)-bitartrate, histamine dihydrochloride, and serotonin hydrochloride were purchased from Sigma-Aldrich Chemie GmbH (Taufkirchen, Germany). All other chemicals were of the highest purity grade commercially available. Deionized water was used throughout the experiments. Stock solutions were freshly prepared daily.

## Results

### Left atrial preparations

**Fig. 2:**
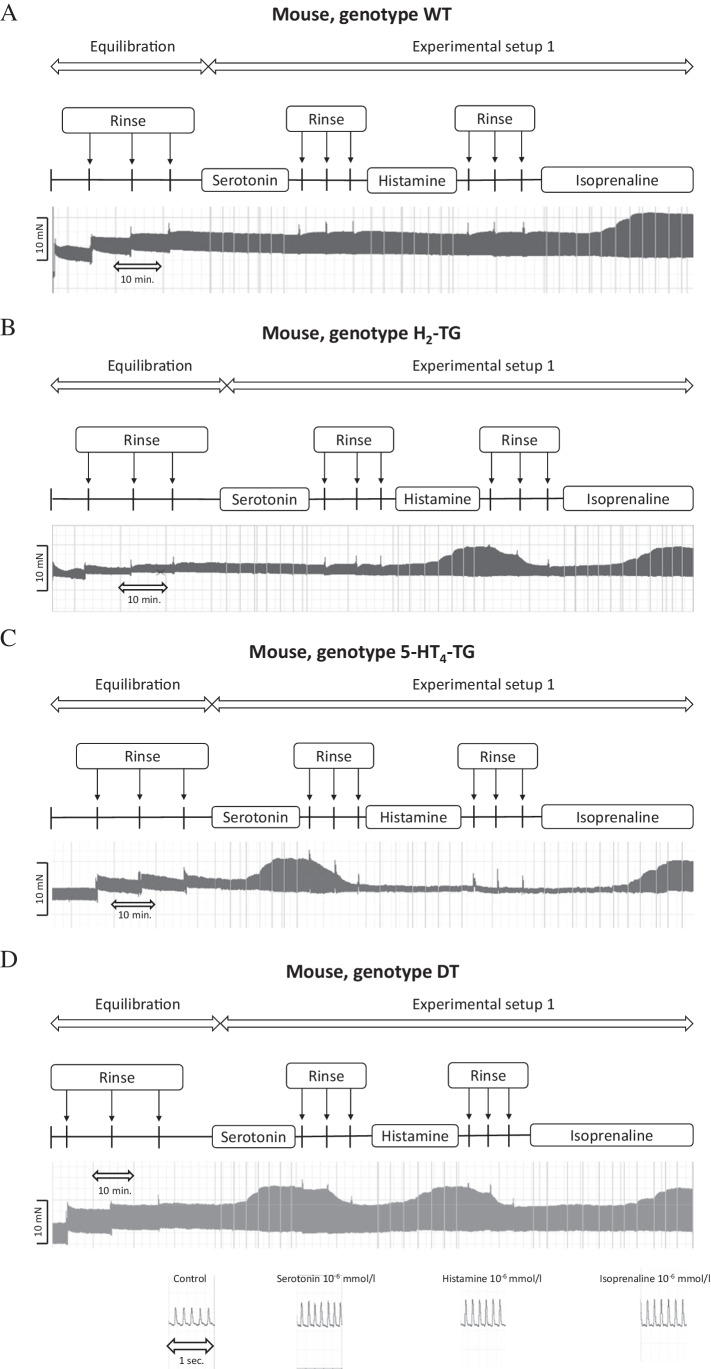

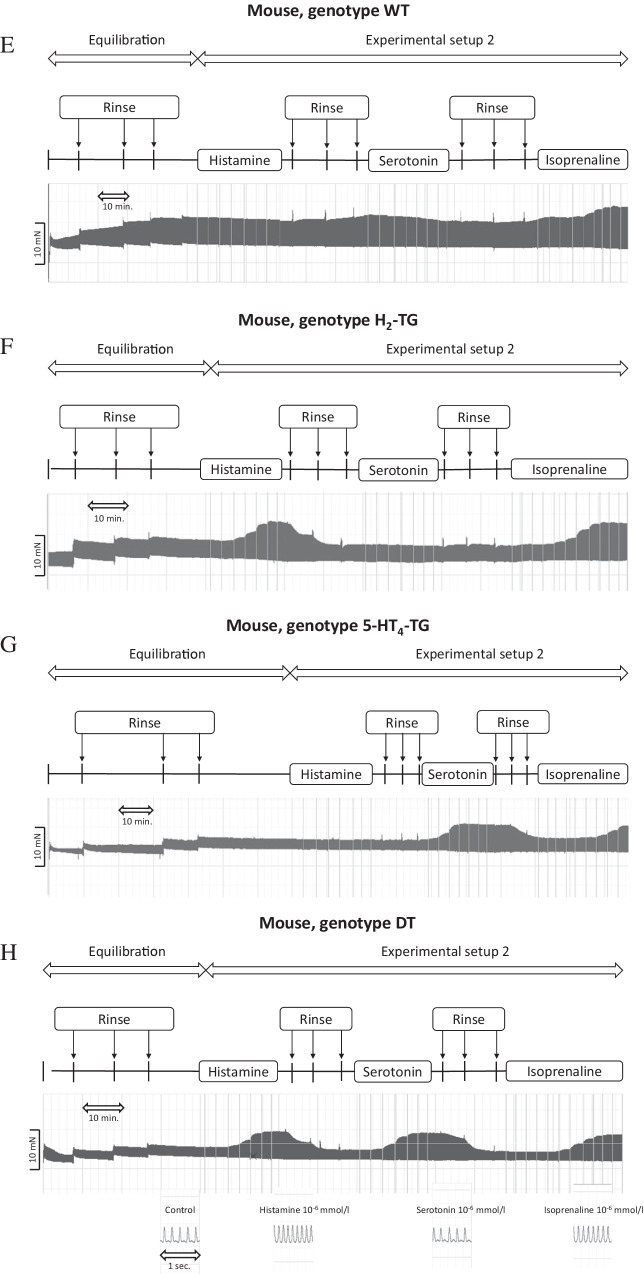
Original recordings of cumulative concentration response curves to histamine or serotonin or isoproterenol in electrically paced left atrial preparations are presented in the upper original recordings. Increases in the amplitude of the recording indicate the inotropic responses to sequentially applied serotonin, then wash out, the histamine, then wash out then isoproterenol. For comparison, at higher temporal resolution, the single spontaneous contractions of right atrial preparations are also recorded. Please note that on the left hand side, the initial beating rate under control conditions (pre-drug value) and then beating rates at the end of the concentrations response curves to sequentially applied serotonin, then wash out, then histamine, then wash out then isoproterenol are presented. In right atrial preparations, the increase in spontaneous beating rate of the three drugs applied is noteworthy. In these typical experiments, concentration response curves of cumulatively applied serotonin (at the indicated concentrations in M) on force of contraction were recorded (**A**–**D**). After complete wash out (= rinse) of histamine effects, serotonin was cumulatively applied, and after another washout, isoproterenol cumulatively was applied. In these DT preparations, both histamine and serotonin exerted positive inotropic effects. Ordinates: force of contraction in mN. Time scales are given by horizontal bars. In half of the experiments, order of the drug addition was reversed: first histamine, then serotonin, then isoproterenol (**E**–**H**)

**Fig. 3:**
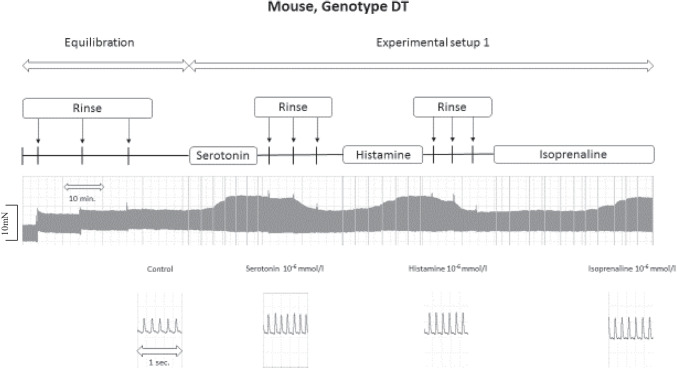

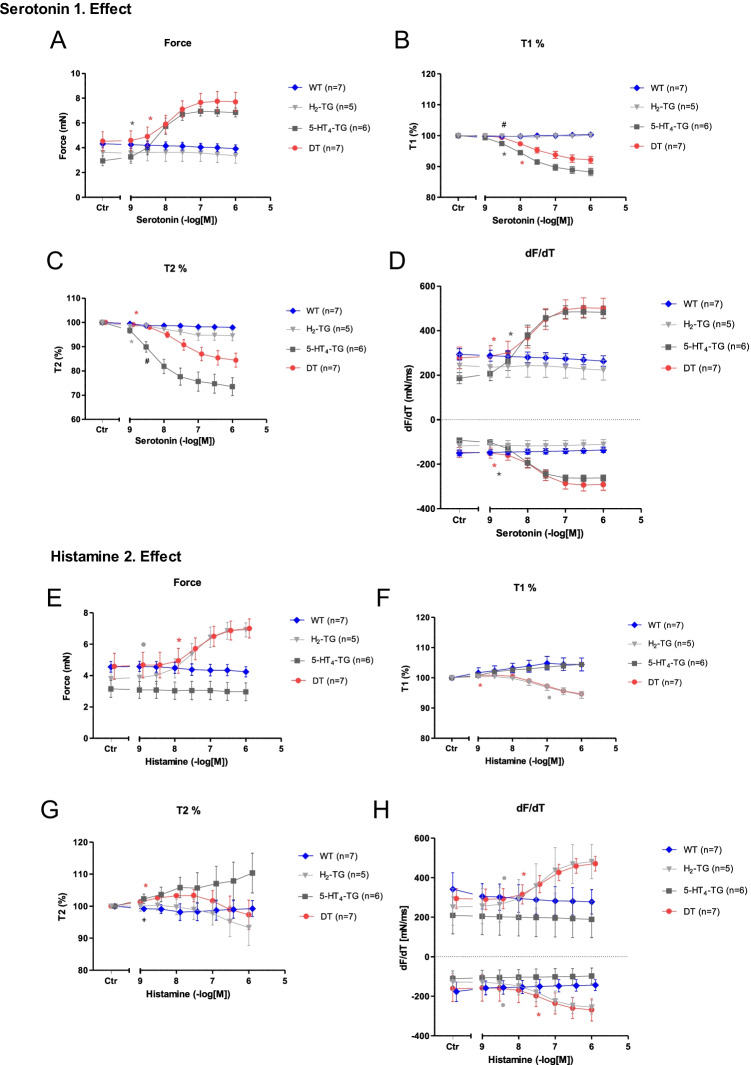
First effect of serotonin and second effect of histamine on force of contraction (**A**, **F**), on change in force of contraction (**B**, **G**), time of contraction (**C**, **H**), and time of relaxation in % of pre-drug value (**D**, **I**) or maximum and minimum of first derivative with respect to force of contraction (**E**, **J**) in isolated electrically driven (1 Hz) atrial preparations from H_2_-TG, 5-HT_4_, DT (5-HT_4_-x H_2_-TG), and wild type (WT) mice. The results of experiments initially stimulated with serotonin. The basic design was as in Fig. 2. Ordinates: force of contraction in mN, change in force of contraction, force in pre-drug values, time in % or pre-drug value and dF/dt in nN/ms. Abscissae: drug concentration shown as negative molar logarithm. Number in brackets indicated number of animals studied. *,, and ○ indicate first significant difference (*P* < 0.05) vs. Ctr (= predrug value) or WT

**Fig. 4:**
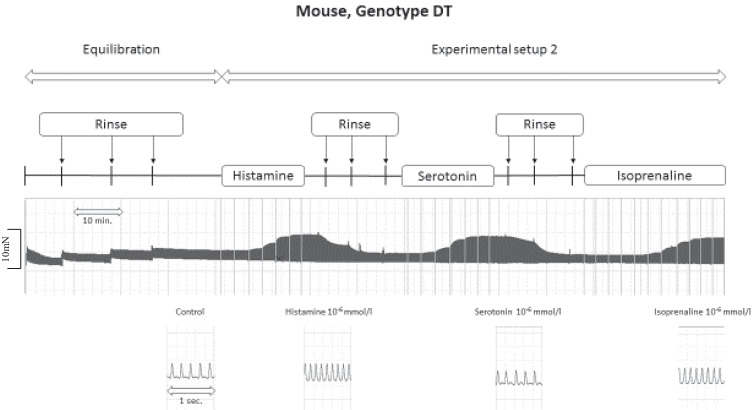

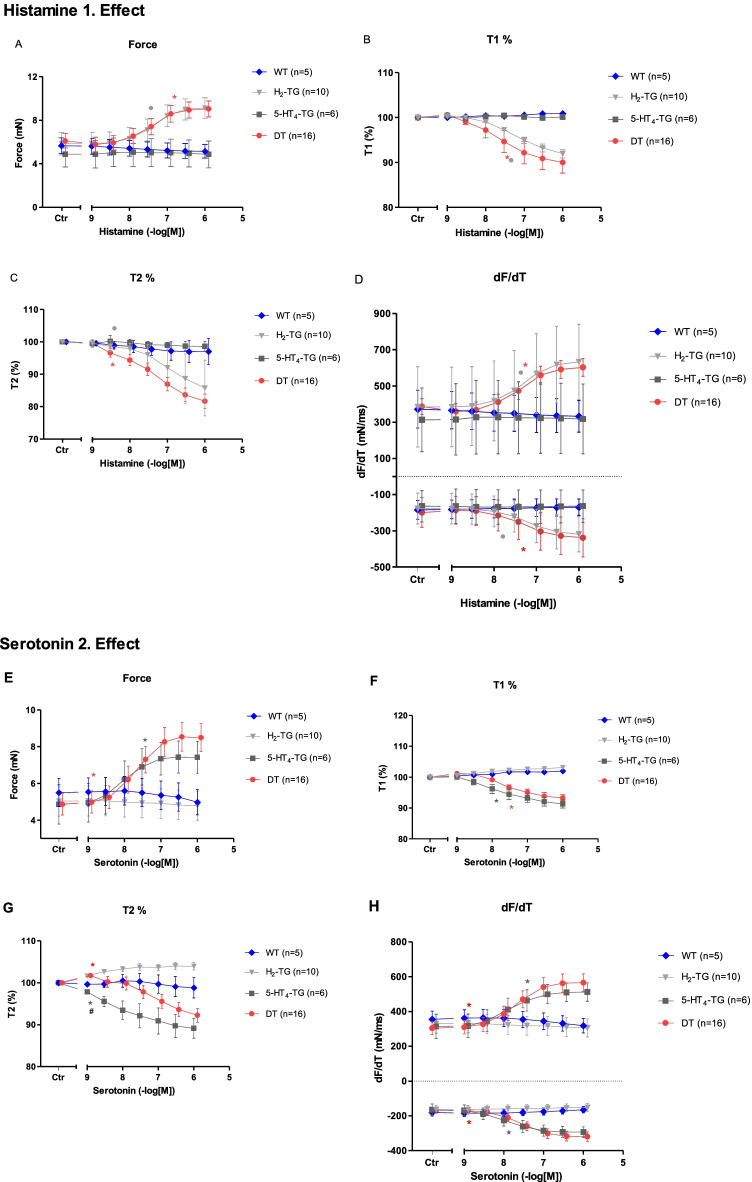
First effect of histamine and second effect of serotonin on force of contraction (**A**, **F**), on change in force of contraction (**B**, **G**), time of contraction (T1: **C**, **H**), and time relaxation in % of pre-drug value (T2: **D**, **I**) or maximum and minimum of first derivative with respect to force of contraction (**E**, **J**) in isolated electrically driven (1 Hz) atrial preparations from H_2_-TG, 5-HT_4_, DT (5-HT_4_-TG x H_2_-TG), and wild type (WT) mice. The results of experiments initially stimulated with histamine. The basic design was as in Fig. 2. Ordinates: force of contraction in mN, change in force of contraction, force in pre-drug values, time in % or pre-drug value and dF/dt in nN/ms. Abscissae: drug concentration shown as negative molar logarithm. Number in brackets indicated number of animals studied. *,, and ○ indicate first significant difference (*P* < 0.05) vs. Ctr (= predrug value) or WT

As seen in the original recordings (Fig. 2) and summarized in Fig. 3, histamine elicited a concentration-dependent PIE in isolated electrically stimulated (1 Hz) left atrial preparations from DT and H_2_-TG mice (Table 1), but histamine did not elicit a PIE in WT and 5-HT_4_-TG preparations (Figs. 2, 3A, B, and C). Likewise, histamine increased the absolute values of the dF/dt_max_ and dF/dt_min_ (Fig. 3D) and decreased the time to peak tension (Fig. 3B) and histamine decreased the time of relaxation (Fig. 3C) in H_2_-TG and DT preparations, but not in 5-HT_4_-TG and WT preparations. Moreover, if histamine was given initially (see Fig. 2 for design) and the results are compared with the effect of histamine (Fig. 4E–H) given after an initial treatment with 5-HT, the PIEs in the DT and H_2_-TG preparations, which originally showed very similar effects, were shifted to higher concentrations of histamine: this suggests a desensitization of H_2_-histamine receptors (Fig. 4B and 4C). More specifically, the potency of histamine was similar in H_2_-TG and DT in the first effect (Fig. 3A, Table 1). In contrast, the potency of histamine was similar in H_2_-TG and DT in the second effect (Fig. 4E, Table 1).

**Table 1:** EC_50_ values

Figure	Histamine I. Effect				Figure	Serotonin II. Effect			
	-log EC_50_		-log IC_50_			-log EC_50_		-log IC_50_	
	DT	H_2_-TG	DT	H_2_-TG		DT	5-HT_4_-TG	DT	5-HT_4_-TG
3A	7.48 ± 0.38	7.34 ± 0.51	-	-	**3F**	7.78 ± 0.32	8.03 ± 0.60	-	-
3B	7.43 ± 0.17	7.35 ± 0.20	-	-	**3G**	7.78 ± 0.17	8.03 ± 0.24	-	-
3C	-	-	7.61 ± 0.33	7.21 ± 0.13	**3H**	-	-	7.50 ± 0.19	7.87 ± 0.25
3D	-	-	7.46 ± 0.18	7.00 ± 0.21	**3I**	-	-	7.16 ± 0.30#	8.29 ± 0.38
3E	7.41 ± 0.34	7.30 ± 0.14	7.35 ± 0.28	7.19 ± 0.35	**3 J**	7.71 ± 0.29	7.98 ± 0.49	7.66 ± 0.26	7.96 ± 0.40
5A	7.18 ± 0.28	7.78 ± 0.28	-	-	**5B**	7.09 ± 0.26	7.40 ± 0.26	-	-
	**Serotonin I. Effect**					**Histamine II. Effect**			
	DT	T-HT_4_-TG	DT	5-HT_4_-TG		DT	H_2_-TG	DT	H_2_-TG
4A	7.93 ± 0.37	8.28 ± 0.19	-	-	**4F**	7.41 ± 0.51	7.50 ± 0.30	-	-
4B	7.93 ± 0.16	8.28 ± 0.15	-	-	**4G**	7.42 ± 0.23	7.50 ± 0.18	-	-
4C	-	-	7.69 ± 0.18	7.96 ± 0.11	**4H**	-	-	7.00 ± 0.14	7.06 ± 0.25
4D	-	-	7.67 ± 0.22#	8.35 ± 0.19	**4I**	-	-	6.08 ± 1.63 +	6.64 ± 0.43
4E	7.89 ± 0.33	8.21 ± 0.18	7.80 ± 0.26	8.14 ± 0.15	**4 J**	7.35 ± 0.43	7.44 ± 0.31	7.25 ± 0.35	7.34 ± 0.26
5C	8.13 ± 0.36	7.37 ± 0.34	-	-	**5D**	7.29 ± 0.23	7.16 ± 0.17	-	-
	**Serotonin**					**Histamine**			
	-log EC_50_		-log IC_50_			-log EC_50_		-log IC_50_	
6	8.21 ± 0.27		-			-		6.67 ± 0.53	
7B	6.44 ± 0.33		-			8.54 ± 0.42		-	
7C	-		6.29 ± 0.68			8.79 ± 2.22		-	
7D	6.24 ± 0.35		6.17 ± 0.48			8.61 ± 0.36		8.54 ± 0.37	

^#^ indicate significant difference (*P* < 0.05) 5-HT_4_-TG vs. DT; + indicate significant difference (*P* < 0.05) H_2_-TG vs. DT

As seen in the original recordings (Fig. 2) and summarized in Fig. 4, in the second series of experiments, initially given 5-HT induced a concentration-dependent PIE in isolated electrically stimulated (1 Hz) left atrial preparations from 5-HT_4_-TG and DT mice (Fig. 4A) (Fig. 2C, Table 1), but 5-HT exerted no contractile effect in WT and H_2_-TG preparations (Fig. 4A). Likewise, 5-HT increased the absolute values of the dF/dt_max_ and dF/dt_min_ (Fig. 4D) and concomitantly decreased the time to peak tension (Fig. 4B) and the time of relaxation (Fig. 4C) in the atrial preparations from H_2_-TG and DT mice, but not in atrial preparations from 5-HT_4_-TG and WT mice. Moreover, if 5-HT was given first (see Fig. 2 for design) and the effect was compared with the effect of 5-HT (Fig. 4A) after initial treatment with histamine, the PIE in the DT and H_2_-TG preparations, which initially occurred at different EC_50_ values (Fig. 3E), were shifted to similar EC_50_ values (Table 1). In other words, 5-HT was more potent to increase force of contraction in 5-HT_4_-TG than in DT when 5-HT was given as the first effect (Fig. 4A, Table 1). In contrast, 5-HT appeared similarly potent in 5-HT_4_-TG than in DT when 5-HT was added in the second effect (Fig. 3E, Table 1).

### Right atrial preparations

**Fig. 5:**
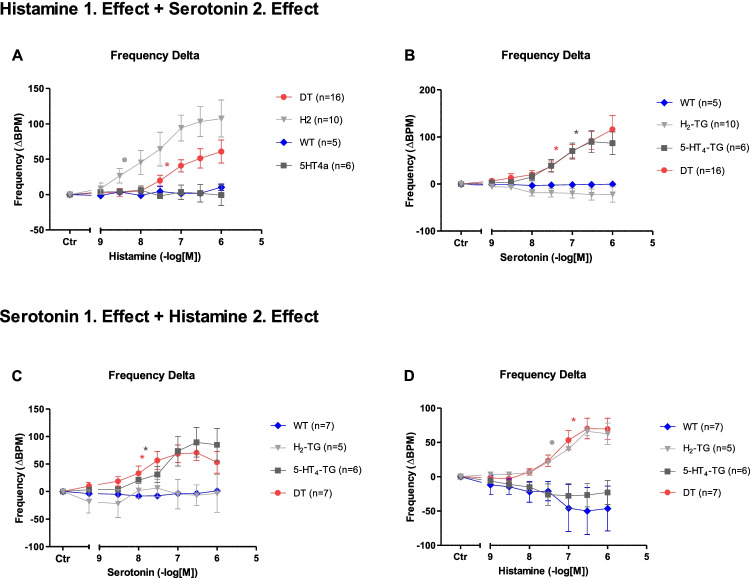
Beating rate in isolated spontaneously beating right atrial preparations from H_2_-TG, 5-HT_4_-TG, DT (5-HT_4_-TGxH_2_-TG), and wild type (WT) mice. The results of experiments initially stimulated with **A** histamine (first effect), secondly stimulated with **B** serotonin (second effect) or initially stimulated with **C** serotonin (first effect), secondly stimulated with histamine (second effect) are depicted. The basic design was as in Fig. 2. Ordinate: Change in beats per minute (bpm). Abscissae: drug concentrations in negative molar logarithm. and ○ indicate first significant difference (*P* < 0.05) vs. Ctr or WT

Similarly to the observations in left atrial preparations described above, histamine led to a concentration-dependent PCE (Table 1) in right atrial preparations of H_2_-TG and DT mice, as shown in Fig. 5A, but was ineffective to exert a PCE in WT and in 5-HT_4_-TG preparations (Fig. 5A). Histamine displayed more potent and maximal effects in naïve atria from H_2_-TG as compared to DT mice (Fig. 5D). No such difference was observed when the concentration–response curves to histamine were measured after the responses to serotonin had been measured (Fig. 5D).

Similarly, 5-HT elicited a concentration-dependent PCE (Table 1) in right atrial preparations of 5-HT_4_-TG and DT mice (Fig. 5C), but had no effect in WT preparations (Fig. 5C). When histamine was given first, it was more potent in DT than in 5-HT_4_-TG preparations for eliciting a PCE (Fig. 5B, Table 1). However, when histamine followed the 5-HT treatment (see Fig. 2 for the design), the potencies were similar (Fig. 5D, Table 1).

### Interactions

**Fig. 6:**
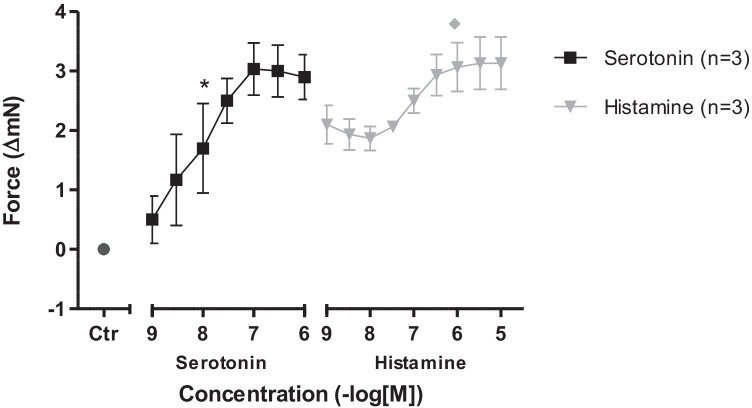
Interaction of serotonin and histamine in mouse. Change in force of contraction in isolated electrically driven (1 Hz) left atrial preparations from DT. The atrial trabeculae were initially stimulated with cumulatively applied serotonin then without washout histamine was cumulatively applied. Abscissae: drug concentrations shown as negative molar logarithm. Number in brackets indicated number of animals studied. and ◊ indicate first significant difference (*P* < 0.05) vs. Ctr (= predrug value) or C_max_ of serotonin

Having established the DT model, we then used this model to look for interactions between the H_2_- and 5-HT_4_-receptors in DT preparations. We first applied 5-HT and again detected a PIE in the left atria (Fig. 6). Thereafter, 5-HT was not washed out as in the experiments reported above, but histamine was additionally applied in the continued presence of 5-HT. We now noted a biphasic effect of histamine: at low concentrations, histamine reduced the force of contraction but at higher concentrations, histamine increased the force of contraction (Fig. 6). The opposite was not the case: in further experiments we first stimulated isolated left atria from DT maximally with 1 µM histamine and then treated the atria in the organ bath with cumulative concentrations of 5-HT. Under these conditions 5-HT (1 nM to 1 µM) failed to decrease force of contraction (*n* = 5, data not shown).

### Human right atrial preparations

**Fig. 7:**
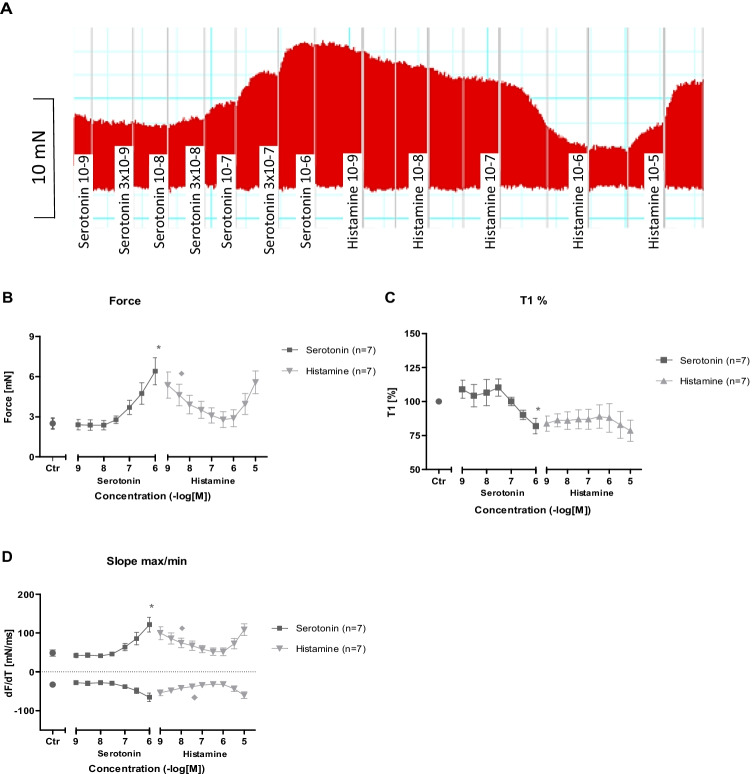
Interaction of serotonin and histamine in humans. Force of contraction (**b**), time to peak tension (t1%; **C**), maximum and minimum of first derivative with respect to force of contraction (**D**) in isolated electrically driven (1 Hz) atrial preparations patients. The atrial trabeculae were initially stimulated with cumulatively applied serotonin then without washout histamine was cumulatively applied. A typical original recording is seen in **A**. Ordinates: force of contraction in mN, time in % of pre-drug value and dF/dt in nN/ms. Abscissae: drug concentrations shown as negative molar logarithm. Number in brackets indicated number of preparations out of four patients. and ◊ indicate first significant difference (*P* < 0.05) vs. Ctr (= predrug value) or C_max_ of serotonin

We next translated these findings to humans using human right atrial preparations. As in the DT mouse preparations, pre-stimulation with 5-HT, followed by histamine, first showed a reduction in force of contraction (10 nM histamine) and then at higher concentrations of histamine (1–10 µM histamine; Fig. 7A–D) an increase in force of contraction ensued.

### M-mode echocardiography of mice

**Fig. 8:**
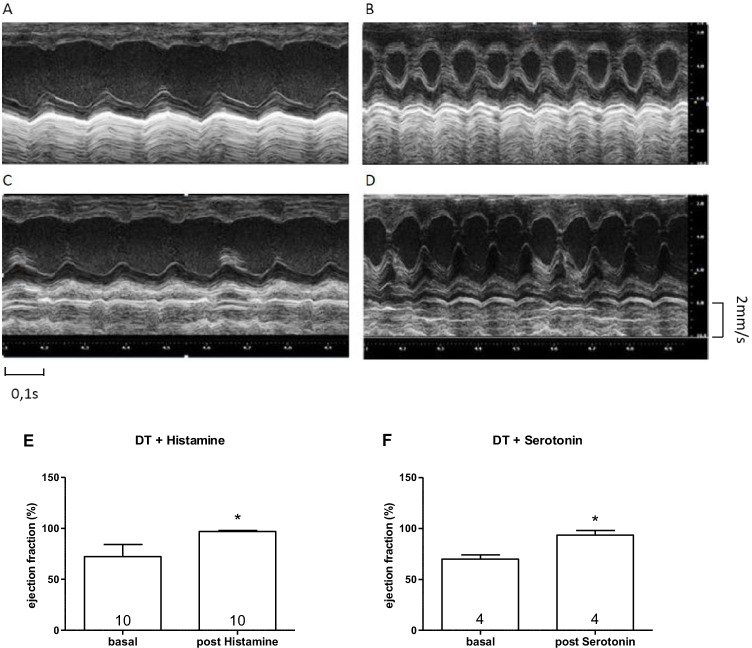
Original M-mode and summarized data of DT before and after injection of histamine (**A**, **B**) or serotonin (**C**, **D**). Ejection fraction in % before and after injection of histamine (**E**) or serotonin (**F**). Numbers in graphs indicate number of animals studied. * indicate a significant difference (*P* < 0.05) vs. basal (pre-drug values)

Similar to our data on the isolated atrium, histamine increased the ejection fraction (EF), an established measurement of left ventricular contractility in living animals, in narcotized H_2_-TG and in narcotized DT mice, but not in narcotized 5-HT-TG and in narcotized WT mice. Likewise, 5-HT increased the EF in narcotized 5-HT-TG and narcotized DT mice (Fig. 8).

### Isolated perfused heart according to Langendorff

Analogous to our data on isolated atrium, histamine increased the beating rate and the left ventricular force of contraction and raised the absolute values of the rate of tension development (dF/dt) in Langendorff-perfused H_2_-TG and DT mouse hearts, but not in 5-HT-TG and WT mouse hearts. Likewise, 5-HT increased the beating rate and dF/dt_max_ in Langendorff-perfused 5-HT-TG and DT mouse hearts, but not in H_2_-TG and WT mouse hearts. As a control, isoprenaline increased the beating rate and dF/dt in Langendorff-perfused H_2_-TG, 5-HT_4_-TG, DT, and WT mouse hearts (Table 2).

**Table 2:** Effects of 1 µM 5-HT and 1 µM histamine after 5 min of application on developed force, and its first derivative vs. time. In each condition, five different DT were studied. At the end of 5 min, the hearts were rapidly frozen and used for western blots

	Ctr	1 μm 5-HT	Ctr	1 μm histamine
mN	12.7 ± 1.34	21.65 ± 1.78 *	13.3 ± 1.29	18.8 ± 1.43 *
dF/dtmax mN/s	356 ± 36.1	865 ± 68.8 *	444 ± 35.4	735 ± 48.0 *
dF/dtmin mN/s	317 ± 41.8	886 ± 78.5 *	439 ± 57.4	759 ± 48.0 *

^*^ indicates significant differences (*P* < 0.05) vs. pre-drug values (Ctr)

### Phosphorylation state of phospholamban

**Fig. 9:**
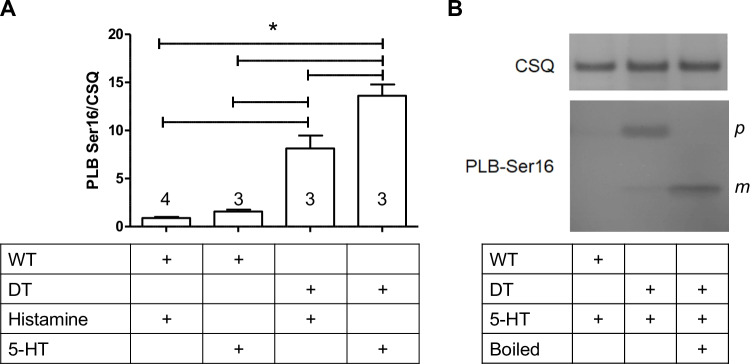
Western blot data for phospholamban (PLB) phosphorylation on serine 16 of wild type (WT) or double transgenic (DT) mice in the presence of 1 µM serotonin (5-HT) or 1 µM histamine, respectively. **A** Quantification of PLB-Ser16 phosphorylation normalized to calsequestrin (CSQ) expression. Numbers in bars indicate number of animals studied. * indicate a significant difference (*P* < 0.05). **B** Original blots: the correct PLB band can be identified by comparing boiled and non-boiled samples because boiling forces PLB from its pentameric (*p*) form to the monomeric (*m*) form

Similar to our contractile data from isolated atria and left ventricles (= Langendorff-perfused hearts), histamine increased phospholamban phosphorylation (Fig. 9) on serine 16 in isolated perfused hearts from H_2_-TG and DT mice, but not from 5-HT-TG and WT mice. Likewise, 5-HT increased phospholamban phosphorylation on serine 16 in ventricles from 5-HT_4_-TG and DT mice, but not from H_2_-TG and WT mice (Fig. 9).

### Receptor expression

**Fig. 10:**
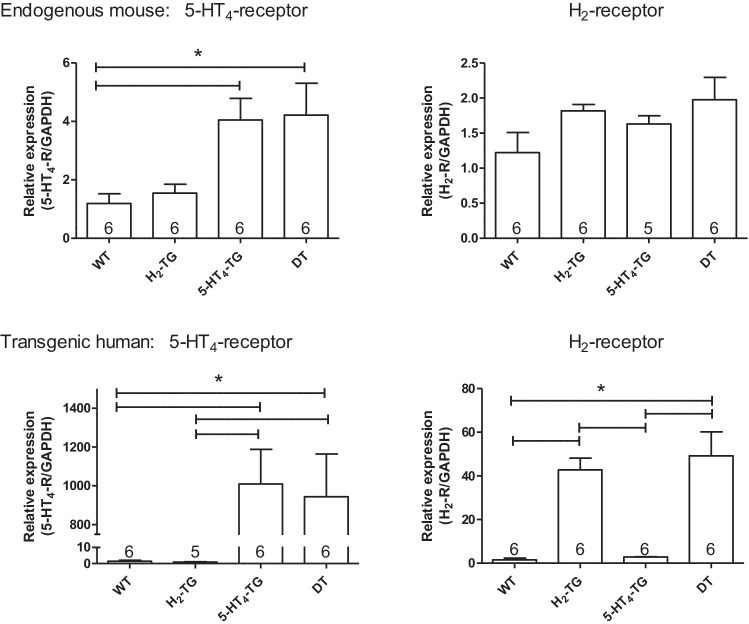
Quantitative polymerase chain reaction for H_2_- and 5-HT_4_ receptors in hearts of H_2_-TG, 5-HT_4_,-TG, DT (5-HT_4_-x H_2_-TG), and wild type (WT) mice. Data are presented in bar diagrams. Numbers in bars indicate number of animals studied. Please note that the upper bar diagrams indicates the expression of the endogenous mouse receptors, whereas the lower bar diagrams represent the transgenically expressed human receptors in the mouse hearts. Please also note the different scales in the ordinates. The expression of endogenous mRNA for the endogenous mouse H_2_- and 5-HT_4_ receptors is remarkably low (upper bars), whereas in the transgenic heart to appropriated transgene (the human H_2_- and 5-HT_4_ receptors) are heavily expressed in the approparitate monotransgenic and the double transgenic hearts. No relevant expression (back ground signal) of the human H_2_-receptors is found in 5-HT_4_-TG and no relevant expression of the human 5-HT_4_ receptors is found in H_2_–TG. * indicate a significant difference (*P* < 0.05)

As seen in Fig. 10 (bottom), the transgenic human H_2_-receptor and the transgenic human 5-HT_4_-receptors are very highly expressed (compare different scaling of the ordinates in Fig. 10 top and bottom) in H_2_-TG, or 5-HT-TG and DT. The endogenous mouse 5-HT_4_-receptor is substantially increased in its expression in 5-HT_4_-TG and DT. In contrast the expression of mouse H_2_-receptors is low compared to mouse 5-HT_4_-receptor and is not significantly different between the genotypes tested here (Fig. 10, top) (Table 3).

**Table 3:** Primers used for the quantitative determination of mRNA coding for endogenous or exogenous (transgenic human) 5-HT_4_-receptors and H_2_-receptors in hearts of mice

Gene	Primer sequence 5′ → 3
Mouse 5-HT_4_-receptor	For: CAAACTTGATGCTAATGTGAGTTC
	Rev: AAGGCGAGAGACACAATGAA
Mouse H_2_-receptor	For: CGCGTTGCCATCTCTTTGGTCTTT
	Rev: TCGTTGACCTGCACTTTGCACTTG
GAPDH	For: CCAGCCTCGTGTAGAC
	Rev: ATGGCAACAACTTTGC
Myosin heavy chain (MHC)	For: ACCCTTACCCCACATAGACC
Human 5-HT_4_-receptor	Rev: AAACACCTCCAAATCC
Human H_2_-receptor	Rev: AGCAGGTCAGTGATAGCCAA

## Discussion

As expected for the DT mice that overexpress both the 5-HT_4_- and H_2_-receptors, 5-HT and histamine elicited a PIE and a PCE. We noticed a mechanical effect in all regions of the heart. For instance, histamine and serotonin (5-HT) stimulated the left atrium and the left ventricular force, as measured in Langendorff-perfused hearts, as well as the left ventricular wall motion in the living animal, as assessed by echocardiography. Likewise, the PCE response to histamine and 5-HT was not only seen in isolated right atria, but also in isolated perfused hearts. Hence, we have succeeded in overexpression in the left atrium, right atrium and left ventricle based on our functional data.

In addition, the signal transduction in DT seems at least consistent with the signal transduction in the human heart: we noted an increase in phospholamban phosphorylation in DT preparations after histamine or 5-HT application. This is consistent with our earlier work, where we measured an elevation of phospholamban phosphorylation in the human heart by histamine via H_2_-receptors and by 5-HT via 5-HT_4_-receptors (Gergs et al. 2017, 2009). Increases in phospholamban phosphorylation after application of histamine or 5-HT are also in line with our mechanical findings: it is generally accepted that phospholamban phosphorylation, at least in part, causes an increased rate of tension development and a shortening of the time of relaxation because phosphorylated phospholamban enhances Ca^2+^ uptake into the sarcoplasmic reticulum (Fig. 1 and Haghighi et al. 2014).

The effects of histamine or 5-HT were also noted in living mice by echocardiography. Hence, we would argue that we have generated a new double transgenic mouse line that is able to recapitulate some effects of histamine and 5-HT that are known to occur in the human heart via active H_2_ receptors and active 5-HT_4_ receptors. We successfully expressed the human receptor sequences in our model.

Hence, this mouse model should provide more insights into human receptor pharmacology and allow the testing of hypotheses that can then be confirmed in human isolated cardiac preparations.

Our experiments showed that the order of treatment did not qualitatively matter: if histamine was given first and then 5-HT or whether 5-HT was given first and then histamine, the resulting contractile responses were qualitatively the same. The effects of histamine on force of contraction were superimposable in the DT and H_2_-TG mice. By contrast, 5-HT elicited a less potent increase in the force of contraction of DT mice than of 5-HT_4_-TG mice.

Hence, the co-expressed 5-HT_4_ and H_2_-receptors could interact in an inhibitory fashion. Dimeric heteromeric G-protein coupled receptors are known to exist, and the dimerization in other receptors leads to loss of potency (Rukavina Mikusic et al. 2020). Conversely, the density of 5-HT_4_ is lower in 5-HT_4_-TG than in DT mice (Fig. 9).

We also noticed a heterologous desensitization. We had shown previously that 5-HT shows homologous desensitization in 5-HT_4_-TG mice (Gergs et al., 2017), but we noted more desensitization in the H_2_–receptor mediated actions and most in the 5-HT_4_ receptor-mediated action. Consistent with this explanation, we have reported pronounced homologous desensitization in 5-H4-TG mice and hardly measurable desensitization in H_2_-TG mice (Gergs et al. 2017,  2019b).

We used the DT mice in the next step to study whether a functional interaction exists between human H_2_- and human 5-HT_4_-receptors in the atrium. Somewhat unexpectedly, we noted that when we did not wash out 5-HT (as was done in Fig. 2), the added histamine was not inactive (we had assumed that 5-HT should have activated the force maximally, so a further increase by another cAMP coupling receptor was not expected), as it decreased the force of contraction. We suggest this as the first confirmation of the validity of our DT mice as a model for the human atrium, as we noted exactly the same negative inotropic effect of small concentrations of histamine in the continued presence of maximally active (with respect to contractility) 5-HT in the human atrium. We now speculate that this inhibitory effect of histamine in the human heart may represent a protective mechanism, since 5-HT can reach very high concentrations in the human atrium after thrombosis and has been suggested to maintain or induce cardiac arrhythmias via stimulation of cardiac 5-HT_4_ receptors (Kaumann and Levy 2006).

Interestingly, previous work has shown that, during stimulation of protein kinase C with phorbol esters, stimulation of β_1_-adrenoceptors with isoprenaline can inhibit the current through L-type Ca^2+^ channels stimulated by histamine in guinea pig ventricular cardiomyocytes (Belevych et al. 2004) that express H_2_ receptors (Verma and McNeill 1977). Similarly, histamine could block the isoprenaline-induced increase in current through L-type Ca^2+^-channels in guinea pig ventricular cardiomyocytes, and this effect was blocked by H_2_-blockers (but not H_1_-blockers: Belevych et al. 2004). This effect was not seen in cardiomyocytes pretreated with pertussis toxin, which is known to functionally block pathways involving inhibitory GTP-binding proteins (Belevych et al. 2004). These researchers suggested that the antagonistic effects of between isoprenaline and histamine might relate to the observation that both couple via stimulatory GTP-binding proteins to adenylyl cyclase as well as via inhibitory GTP-binding proteins to adenylyl cyclase. Indeed, others have reported that histamine can bind to inhibitory GTP-binding proteins (Kilts et al. 2000). This previous work showed that pertussis toxin pretreated human atrial membranes show an increase in the stimulatory action of histamine on adenylyl cyclase activity when compared to samples not previously treated with pertussis toxin (Kilts et al. 2000). We hypothesize that a similar mechanism might also explain the interaction between serotonin acting on 5-HT_4_ receptors and histamine treatment in DT mice, as 5-HT is also known to bind to inhibitory GTP-binding proteins (Kilts et al. 2000). This is an interesting explanation for the findings of our present study but still needs experimental confirmation. Another explanation for our findings in the human atrium might be found in the work of Levi and colleagues (Guo et al. 1984). They observed interestingly in electrically stimulated but also spontaneously beating right atrial preparations from surgical patients a negative inotropic effect of histamine that was blocked by mepyramine and thus probably mediated by H_1_-receptors whereas higher concentrations of histamine led to positive inotropic effect that were cimetidine sensitive and thus probably H_2_-receptor mediated. Thus, future studies might test whether the negative inotropic effect we noted in the presence of serotonin in human atrial preparations might be H_1_-receptor mediated.

In summary, we demonstrate for the first time the possibility of functionally co-overexpressing human H_2_- and human 5-HT_4_-receptors in the same mouse heart. We noted an inhibitory interaction between histamine and 5-HT in DT mouse hearts as well as in human atrial muscle strips. We speculate that this interaction can dampen the detrimental effects (like arrhythmias) of 5-HT in human hearts and may act as a brake on the cardiac actions of 5-HT under pathophysiological conditions.

## Supplementary Information

Below is the link to the electronic supplementary material.

Supplementary file1 (PDF 59 KB)

Supplementary file2 (PZF 1341 KB)

Supplementary file3 (PZF 1374 KB)

Supplementary file4 (PZF 1262 KB)

Supplementary file5 (PZF 1275 KB)

Supplementary file6 (PZF 1205 KB)

Supplementary file7 (PZF 178 KB)

Supplementary file8 (PZF 781 KB)

Supplementary file9 (PZF 250 KB)

Supplementary file10 (PZF 2238 KB)

Supplementary file11 (PZF 1474 KB)
