# Correction to: Functional interaction of H_2_‑receptors and 5-HT_4_‑receptors in atrial tissues isolated from double transgenic mice and from human patients

**DOI:** 10.1007/s00210-021-02167-2

**Published:** 2021-11-05

**Authors:** Joachim Neumann, Denise Schwarzer, Charlotte Fehse, Rebecca Schwarz, Margareta Marusakova, Uwe Kirchhefer, Britt Hofmann, Ulrich Gergs

**Affiliations:** 1 Institut für Pharmakologie und Toxikologie, Martin-Luther-Universität Halle-Wittenberg, Medizinische Fakultät, Magdeburger Str. 4, 06112 Halle, Germany; 2 Department of Pharmacology and Toxicology, Faculty of Pharmacy, Comenius University in Bratislava, Bratislava, Slovakia; 3 Institut für Pharmakologie und Toxikologie, Westfälische Wilhelms-Universität, Medizinische Fakultät, Domagkstr. 12, 48149 Münster, Germany; 4 Cardiac Surgery, Martin-Luther-Universität Halle-Wittenberg, Medizinische Fakultät, 06097 Halle, Germany

**Correction to** **:** **Naunyn–Schmiedeberg’s Archives of Pharmacology**

https://doi.org/10.1007/s00210-021-02145-8

The authors regret that the Fig. 7A contains only a low quality screenshot of the original recording with additional content not intended for the publication. In the first draft, this screenshot acted as placeholder that should be replaced. During figure preparation, it was unfortunately forgotten to insert the final version of the original recording.

In order to meet the high standard of Naunyn-Schmiedebergs Archives of Pharmacology and to ensure data protection, we would like to perform a corrigendum of Fig. 7A, which would only affect the graphical presentation of the original recording and not the scientific statement. The revised Fig. 7 is shown below.
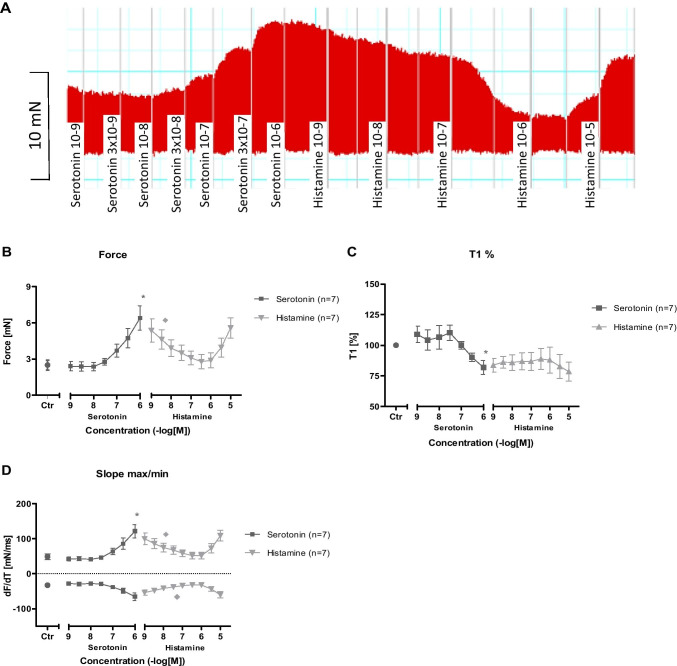


We sincerely apologize for any inconvenience.

