# Supplementary figures and images for: Functional interaction of H2-receptors and 5HT4-receptors in atrial tissues isolated from double transgenic mice and from human patients
Source: Naunyn Schmiedebergs Arch Pharmacol. 2021 Sep 25;394(12):2401–18. doi: 10.1007/s00210-021-02145-8 (PMC8592968; doi:10.1007/s00210-021-02145-8)

CSQ ➔

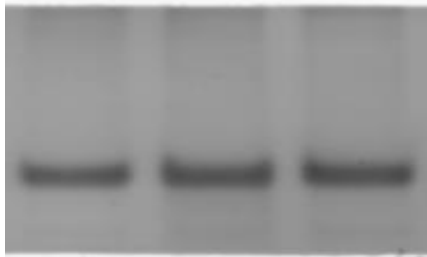

PLB-  
Ser16-P

pentameric ➔

monomeric ➔

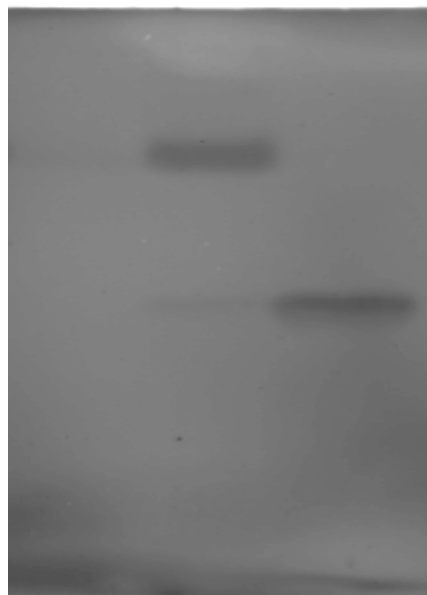

|        |   |   |   |
|--------|---|---|---|
| WT     | + |   |   |
| DT     |   | + | + |
| 5-HT   | + | + | + |
| Boiled |   |   | + |

Supplement: Supplementary file 1 — Supplementary file1 (PDF 59 KB) [file 210_2021_2145_MOESM1_ESM.pdf]
